# Barriers to and facilitators of the development and utilization of context appropriate evidence based clinical algorithms to optimize clinical care and patient outcomes in the Tikur Anbessa emergency department: a multi-component qualitative study

**DOI:** 10.1186/s12913-019-4008-2

**Published:** 2019-03-20

**Authors:** Lisa M. Puchalski Ritchie, Finot Debebe, Aklilu Azazh

**Affiliations:** 1 Department of Medicine, University of Toronto, Toronto, Canada; 2 Department of Emergency Medicine, University Health Network, Toronto, Canada; 3 Knowledge Translation Program, Li Ka Shing Knowledge Institue, St. Michael’s Hospital, 30 Bond Street, Toronto, ON M5B 1W8 Canada; 4 Department of Emergency Medicine and Critical Care, Addis Ababa University School of Medicine, Addis Ababa, Ethiopia

**Keywords:** Emergency medicine, Evidence-Based Clinical Algorithms, Barriers, Facilitators

## Abstract

**Background:**

Evidence-based clinical algorithms (EBCA) are knowledge tools to promote evidence use by codifying evidence into action plans to facilitate appropriate care. However, their impact on process and outcomes of care varies considerably across practice settings and providers, highlighting the need for tailoring of both these knowledge tools and their implementation strategies to target end users and the setting in which EBCAs are to be employed.

Leadership at the Tikur Anbessa Specialized Hospital emergency department (TASH-ED) in Addis Ababa, Ethiopia identified a need for context-appropriate EBCAs to improve evidence uptake to mitigate care gaps in this high volume, high acuity setting. We aimed to identify barriers and facilitators to utilization of EBCAs in the TASH-ED, to identify priority targets for development of EBCAs tailored for the TASH-ED context and to understand the process of care in the TASH-ED to inform implementation planning.

**Methods:**

We employed a multi-component qualitative design including: semi-structured interviews with TASH-ED clinical, administrative and support services staff, and Toronto EM physicians who had worked in the TASH-ED; direct observation of the process of care in TASH-ED; document review.

**Results:**

Although most TASH-ED participants reported an awareness of EBCAs, they noted little or no experience using them, primarily due to the poor fit of many EBCAs to their practice setting. All participants felt that context-appropriate EBCAs were needed to ensure standardized and evidence-based care and improve patient outcomes for common ED presentations. Trauma, sepsis, acute cardiac conditions, hypertensive emergencies, and diabetic keto-acidosis were most commonly identified as priorities for EBCA development. Lack of medication, equipment and human resources were identified as the primary barriers to use of EBCAs in the TASH-ED. Support from leadership and engagement of stakeholders outside the ED where EBCAs were believed to be less well accepted were identified as essential facilitators to implementation of EBCAs in the TASH-ED.

**Conclusions:**

This study found a perceived need for EBCAs tailored to the TASH-ED setting to support uptake of evidence-based care into routine practice for common clinical presentations. Barriers and facilitators provide information essential to development of both context-appropriate EBCAs and plans for their implementation in the TASH-ED.

**Electronic supplementary material:**

The online version of this article (10.1186/s12913-019-4008-2) contains supplementary material, which is available to authorized users.

## Background

Despite the proliferation of medical research in recent decades, healthcare systems globally fail to optimally use evidence to improve patient care and improve both quality of life and mortality [1]. Evidence-based clinical algorithms (EBCA), which include clinical practice guidelines, clinical pathways, order sets, and clinical decision rules [2], provide useful knowledge tools to facilitate incorporation of evidence into routine practice. They accomplish this by “codifying evidence into specific rules or action plans” that can facilitate the delivery of appropriate evidence-based care [2, p 1016]. However, their impact on process and outcomes of care varies considerably across practice settings and providers [2–4], highlighting the need for tailoring of both knowledge tools and their implementation strategies to target end users and the setting in which EBCAs are to be employed [4]. As most EBCAs are developed by and for use in high-income countries, the need for tailoring for low-resource health care settings is likely to be greater. Given the time and cost involved in producing evidence-based knowledge tools, adaptation of high-quality EBCAs has been advocated by knowledge translation experts as a promising approach to avoid duplication of effort and increase applicability of knowledge tools [4]. A key step in the adaptation process is identification of factors at the system, provider and patient levels, operating as barriers to and facilitators of implementation of EBCAs in a given practice setting. In subsequent steps, identified barriers can be addressed through adaptation and tailoring of EBCAs to the local practice context and facilitators optimized to improve implementation. Engagement of stakeholders throughout the process is important to ensuring that issues of applicability, feasibility and sustainability are adequately addressed.

The Tikur Anbessa Specialized Hospital (TASH) is the largest publicly funded tertiary care academic teaching hospital and referral center affiliated with the Addis Ababa School of Medicine. The TASH emergency department (ED) has 20–22,000 ED visits annually, and predominantly manages seriously ill or injured patients, with low acuity cases redirected to appropriate outpatient clinics in the immediate vicinity of the ED during regular hours. It is home of the first emergency medicine (EM) training program in the country, launched in 2010, with 10 EM physician graduates at the time of this study. In view of the small number of emergency medicine-trained physicians, the department is routinely staffed by EM and off-service residents supported by a small number of new EM graduates and consultant non-EM faculty. Given the high volume of high acuity patients managed and limited number of EM consultant physician staff, the TASH-ED leadership identified a need for context-appropriate EBCA to improve uptake of evidence and improve care.

### Study aim

We aimed to identify barriers and facilitators to implementation and utilization of EBCAs in the TASH-ED. Additional objectives were to assess stakeholder perceptions regarding the need for and perceived priority targets for development and implementation of EBCAs tailored for the TASH-ED practice context and to understand the context and process of care in the TASH-ED to inform implementation planning.

## Methods

### Design

Multi-component qualitative study including: stakeholder interviews, direct observation of the process of care and flow of patients through the TASH- ED, and document review.

### Participants

Participants were purposefully selected to represent the range of stakeholders from Tikur Anbessa Specialized Hospital clinical, administrative and essential support services staff who had worked in the TASH-ED for at least 1 month, and University of Toronto affiliated hospital emergency physicians who have worked clinically in the Tikur Anbessa Emergency Department as part of the Toronto Addis Ababa Academic collaboration in emergency medicine (TAAAC EM). TAAAC EM is a collaboration between Adds Ababa and Toronto universities to support the development and implementation of the EM residency program at Addis Ababa University, based at TASH, with emergency medicine faculty from University of Toronto affiliated hospitals providing onsite clinical teaching 3 months each year since the programs’ inception.

A list of potential key informants was developed in consultation with local study team members to represent the range of clinical training (staff physician, residents; masters, bachelor and diploma nurse), primary clinical area (emergency medicine, surgery, internal medicine), and essential support services (pharmacy, radiology, laboratory services, ED management). Snow-ball sampling, which involved asking participants to identify individuals who they felt would provide a unique perspective to the discussion, was subsequently used to identify additional key informants based at TASH. Participants were selected from a list of eligible Toronto participants to represent the range of clinical training, usual practice setting, years of EM experience, and number of months worked in the TASH-ED. Participants identified in the initial TASH and Toronto lists were approached in person or by telephone by the principal investigator (LPR) or a trained research assistant (SB) and invited to participate. Participants identified by snow-ball sampling, were given a brief introduction to the study by the referee and then introduced to the study team. Written informed consent was obtained from all participants. Participants received no remuneration for participation.

### Data collection

Data collection in Ethiopia took place over 2 weeks in July, 2015, and included: a document review, key informant interviews and field observation. Prior to introduction of the study to TASH-ED staff, a search of the clinical, administrative and educational areas of the TASH-ED was undertaken by the Toronto study team, to explore required ED documentation; roles, responsibilities and reporting structure; and any EBCAs available for use in the TASH-ED. Identified paper documents were photographed and electronic documents saved to a USB for analysis. Interviews were conducted by the PI or a trained research assistant using a semi-structured interview guide developed based on the theoretical domains framework (TDF) and previous experience in conducting barrier/facilitator assessments [5, 6] (see Additional file 1). Interviews also sought suggestions for clinical presentations they would most value EBCAs tailored to the TASH-ED context developed for. Interviews were conducted in a private location at TASH at a time convenient to participants. Basic demographic data was collected at the start of each interview, including: background training, current position, years of experience, and for Toronto physicians amount of time spent working in the TASH-ED at the time of the interview. Interviews were conducted in English, with a translator available. Interviews were audio-taped and transcribed verbatim. In order to allow for flexibility to accommodate stakeholder schedules and for observations to reflect times with varying patient volumes and department staffing, field observations and interviews took place concurrently. Observations were conducted by the PI, who is a practicing emergency physician with limited exposure to the TASH-ED prior to the field observation. Observations took place on varying days of the week (commonly busy and less busy days), as well as, at variable times of day (day/evening shifts) and days of the week, for a total of 2 full days. Observations were undertaken in 4 h blocks purposively selected to ensure a comprehensive understanding of changes in patient flow, volumes, and staffing during peak and off-peak hours. A first-year resident based at TASH was present throughout observation to answer any contextual, cultural, or language related questions arising. In order to gain an understanding of the process of care and flow of patients through the emergency department observations were made in: patient arrivals/registration, triage, clinical areas of the department (acute/sub-acute/resuscitation/ED admitted patient area), and support service areas such as radiology, laboratory services, and pharmacy. Field notes were taken during observation. Interviews with Toronto-based physicians were conducted by the PI over 6 weeks in July and August 2015and followed the same interview guide and procedures as for TASH staff. With the exception of one interview during which the recorder failed and hand written notes were taken, as above, interviews were audio taped, and transcribed verbatim.

### Analysis

We used directed content analysis [7], with interviews, as the unit of analysis. NVivo 10 (QSR International Inc., Southport, UK) was utilized to code the data. An initial coding framework based on the theoretical domains framework (TDF) and past experience conducting barrier and facilitator assessments [5, 6] was used. The initial coding framework was expanded based on our preliminary analysis of the interviews. Analysis of interview transcripts occurred in two rounds. First, transcripts were read and coded independently by two study team members (LPR/SB). The coding framework was then revised and applied independently by the same two study team members. Discrepancies were resolved through discussion. Data from field notes were analyzed by the observer with findings discussed with local study team members to ensure accuracy and understanding of observations. Too few relevant documents were identified for analysis.

### Techniques employed to enhance trustworthiness

Several methods were employed during data collection and analysis to enhance the trustworthiness of the study findings. Prior to the start of interviews, participants were informed of the importance of their honest views as these would be used to guide future work to improve patient care and outcomes in their setting. In addition, participants were informed as part of the consent process that their names and interview data would not be accessible by the local study team members, and the findings anonymized so that they could not be identified. Member checks were performed during the interview. Data source triangulation was employed with a wide range of respondents representing all key stakeholder groups within the TASH-ED and from departments providing essential support services, as well as, from ED physicians from University of Toronto affiliated hospitals who had worked in the TASH-ED. Finally, method triangulation [8] was employed in which ED observations and identified documents were considered together with the interview findings to provide a comprehensive understanding of EBCA availability and use in the TASH-ED, and potential barriers to and facilitators of implementation and utilization of context appropriate EBCAs in the TASH-ED.

## Results

### Interview results

#### Characteristics of participants

Twenty-six key informants were interviewed, 18 TASH staff and 8 University of Toronto affiliated hospital emergency physicians. TASH staff participants included a range of clinical, support service and administrative roles, with 5 participants fulfilling combined clinical and administrative roles. TASH participant training backgrounds ranged from off-service first year residents, ED residents from all years of training [1–3], and consultant staff physicians, and diploma, bachelor and masters-trained nurses. Thirteen of 18 (72%) of TASH participants were male, with participants ranging in experience working at TASH from 4 months to > 20 years, and in the TASH-ED from 1 month to > 4 years. All approached agreed to participate. Only one participant opted to have a translator present to provide inline translation as needed.

Toronto based emergency physician participants ranged from early/junior to senior/late career, and worked in a range of clinical settings including: tertiary care academic hospitals, community teaching hospitals and non-teaching hospitals. Half of the participants had worked 1 month in the TASH-ED at the time of the study and half had worked 2 months. Five of 8 (62%) participants were female.

#### Knowledge and beliefs about context appropriate EBCAs

With the exception of TASH and Toronto EM consultants, EBCAs were poorly understood. The most common error in understanding was failure to recognize that EBCAs are evidence based, with examples of non-evidence based protocols or standard operating procedures commonly cited. Experience with EBCAs was also limited among TASH-ED staff, with advanced cardiac and advance trauma life support (ACLS, ATLS) the only commonly cited tools in use among TASH-ED staff. These tools were reported to be used by the majority of staff regularly. All participants felt that EBCAs tailored to the TASH-ED context were needed to improve patient care. Participants felt that EBCAs would standardize and ensure evidence-based care, function as reminder tools to expedite care and ensure essential components of investigation and management were not missed or delayed. Several participants noted that EBCAs could function as advocacy tools, and encourage investment in resources for high impact evidence-based investigations and treatments. Participants prioritized common, high acuity conditions for EBCA development, including: trauma, sepsis, rheumatic heart disease exacerbations, acute coronary syndromes, hypertensive emergencies, and diabetic keto-acidosis. EBCAs addressing uncommon but complex and difficult to manage presentations, such as toxicological emergencies, were identified as important for future EBCA development.

#### Barriers and facilitators identified by interview participants

Although system, provider and patient level barriers and facilitators were specifically and separately queried in interviews, few were identified at the patient level. Many barriers and facilitators were found to interact however, for simplicity of presentation barriers and facilitators are reported within the level in which they primarily operate, see Table 1. Barriers related to lack of resources predominated at the system level including lack of medications, equipment and human resources. Lack of resources were noted to result in lack of fit of most published EBCAs with the TASH-ED context, and therefore limit their applicability and use. Recent investment in EM which is gradually increasing resources and ED leadership to limit delays in care as result of resource shortages were seen as important facilitators to EBCA use. Lack of computer and internet access within the TASH-ED clinical areas were identified as important barriers to EBCA use, with suggestions for paper based EBCAs tailored to the TASH-ED resource and clinical context suggested facilitators to EBCA uptake. Finally, incorporation of EBCAs into policy with consequences for non-use and endorsement of EBCAs by leadership were felt important to EBCA uptake. Barriers identified at the provider level included: lack of interest and poor attitudes toward EBCAs among non-EM trainees, lack of acceptance of EBCAs as an approach to clinical care among non-EM clinical staff, lack of knowledge and skills necessary for EBCA implementation and the challenges associated with changing behavior/habits. With the exception of perceived benefits to both providers and patients, felt to be facilitators of EBCA uptake, the remaining facilitators directly related to identified barriers. For example, while it was recognized that habits are hard to break, the enthusiasm for learning and development of the speciality among EM providers, and potential for lack of “habits” as a relatively new speciality, were felt important facilitators to EBCA use in the TASH-ED. In addition, wide stakeholder engagement, including relevant non-EM consultant staff, in EBCA development and provision of knowledge and skill training were commonly reported suggestions to address identified barriers and facilitate EBCA uptake.

**Table 1:** Barriers to and facilitators of uptake of EBCAs in the TASH-ED

	Barriers/Facilitators	Explanation/Example
Health System Level		
Barriers	Resource constraints.	Lack of consultant level EM physician and nursing staff, high frequency of turnover of trainees rotating through the ED. Lack of medication and equipment, delays in care while patients/families purchase necessary supplies.
	Infrastructure.	Lack of computers and internet connectivity within the TASH-ED, limit accessibility and therefore use EBCAs.
	Fit of the EBCA with the TASH-ED context.	Lack of fit of published EBCAs with the TASH-ED context with respect to both resources and local data.
Facilitators	Strong ED leadership.	ED leadership developed a supply of essential drugs and equipment available for immediate use to reduce delays in urgent care.
	Ministry of health support.	Investment in EM over last decade has and continues to improve EM resources, including material resources and EM trained staff.
	Embedding EBCA into policy.	Embedding the EBCA into the system as hospital policies or guidelines and attaching consequences for non-use, were suggested as facilitators to uptake that had met with success with some previous implementation efforts within the department.
	Endorsement by leadership.	Endorsement by ED and hospital leadership, and the ministry of health, would improve uptake of the EBCA.
	EBCA design and accessibility.	Clear, concise, easy to follow, paper based design, tailored for the practice context in terms of resources and local disease patterns, essential to EBCA uptake.
Provider Level		
Barriers	Acceptance of EBCA approach to clinical care.	While EBCAs reported to be valued by EM staff, participants felt non-EM providers would be resistant to use of EBCAs as a result of differences in work place culture among other clinical departments.
	Attitudes.	Lack of interest/poor attitude among some trainees rotating through the ED.
	Habits.	Practice habits hard to change.
	Knowledge and skills.	Lack of knowledge of EBCA development and basis in evidence and lack of experience with EBCAs common, an important potential barrier to uptake. Lack of knowledge or skills needed for EBCA implementation among non-consultant level staff who provide the majority of hands on care, a key barrier to EBCA uptake.
Facilitators	EM specialty relatively new.	May be fewer habits to break among EM practitioners. Enthusiasm for learning and development of the specialty may be an asset to uptake.
	Perceived benefit of EBCA use.	EBCAs more likely to be used if perceived to benefit the patient and/or provider, ideally both.
	EBCA specific knowledge and skills training.	Provision of appropriate theoretical and practical, knowledge and skills training through didactic and simulation based techniques essential to EBCA uptake and use.
	Wide stakeholder engagement.	Wide stakeholder engagement during development of EBCAs for use in the TASH-ED, particularly inclusion of participants from relevant non-EM departments, suggested to facilitate uptake.
Patient Level		
Barriers	Patient ability to pay.	Many patients lack financial resources to pay for recommended care.
Facilitators	Patient acceptance.	Patients generally accept/agree to provider recommendations.

Relatively few patient level barriers and facilitators were identified, with patient inability to pay for medications and supplies necessary for EBCA based care the only barrier and the general willingness of patients to accept provider recommendations the only facilitator identified.

### Field observation results

Field observation provided both a more in-depth understanding of barriers to and facilitators of uptake and utilization of EBCAs in the TASH-ED, as well as, identified areas of importance for tailoring EBCAs for context and potential strategies for implementation. Chief among these was an appreciation of the high volume of high acuity patients routinely managed and context of care in the TASH-ED, which was recognized as both a barrier to uptake of EBCAs, and reinforced the perceived need among TASH-ED for context appropriate EBCAs to facilitate prompt evidence based care. It was not uncommon for several critically ill and/or injured patients to arrive simultaneously, challenging the ED’s capacity in terms of both space and human resources to triage and begin urgent resuscitation. Due to the small number of ED faculty at the time of this study, the TASH-ED was predominantly staffed by EM and off-service residents in the early years of their training. This finding highlighted the perceived need for quick reference guides for urgent cases to facilitate prompt investigation and initial management, until the senior EM resident and/or staff physician can be consulted. A second important challenge to implementation of EBCAs in the TASH-ED was the physical layout and lack of space. Large numbers of patients were boarded in the ED for long periods of time, leading to crowding in treatment areas and clustering of patients in central spaces and near doorways, making movement of patients and staff through the department, or out of the department to imaging, laboratory services or to the pharmacy, challenging.

Third, both the costs of and cues to pay for laboratory and imaging tests, and medications and supplies, are barriers to utilization of standard EBCAs in the TASH-ED. Opportunities to reduce delays in procuring necessary tests and supplies are important to successful implementation of EBCAs in this context. An example work around implemented in the resuscitation room, where urgent medications and supplies are stored for immediate use and later replaced by the patients family, have been highly successful in reducing delays and suggests opportunities for this approach for other time sensitive conditions. Finally, additional context and process of care issues were also identified as important considerations in selection of strategies for EBCA implementation. Patient charts are paper based and include paper orders/results on non-standard sized forms, making comprehensive test and treat ED order sets inconsistent with standard practice and may represent a challenge to this approach. Visible wall space is limited due to curtains and/or partitions making use of reminder posters less feasible in some areas, and suggests a need for a more readily available reminder formats such as pocket cards, chart attachments or formats for use on personal phones. Computer and internet access are limited within the ED, making computer based formats/application less feasible.

### Document review results

As noted above, despite an extensive search too few relevant documents were identified to allow for qualitative analysis. Nursing staff roles, responsibilities, and reporting structure were well documented and made available to staff. Standard operating procedures (SOPs) for common ED nursing procedures were also well documented, with nursing staff required to read and sign off on SOPs, when changed or when new SOPs were developed. No EBCAs were found to be available in TASH-ED clinical areas. ACLS, ATLS and a neonatal resuscitation guideline were posted in the ED education center classroom (not within the ED), and a small number of EBCAs and non-evidence based ED, critical care, and general medical guidelines were saved to computers in the ED computer lab. It was unclear whether these were saved for reference during clinical work as the computer lab is attached to the ED or whether these documents had been downloaded by residents while preparing presentations as appeared to be the case for some guidelines.

### Triangulated results

Combined barrier and facilitator findings are presented within TDF domains with representative quotes, in Table 2. Although access to and experience with EBCAs was limited, there was a high degree of perceived need for context appropriate EBCAs to improve patient care and outcomes for high burden clinical conditions in TASH-ED. Shortage of human and material resources were identified as the main barriers to EBCAs being used in the TASH-ED. Other reported and observed barriers include high turnover of trainees rotating through the emergency department, lack of physical space making movement within and through the ED challenging, and issues with process of care with respect to accessing medications and supplies, lack of access to and fit of published EBCAs with the TASH-ED context and resistance to use of EBCAs from consultant services. In terms of facilitators, wide engagement of both TASH-ED staff and consultant services were felt essential to successful implementation of EBCAs in the TASH-ED. Other facilitators include strong leadership in the ED, and interest in and a recognized need for EBCAs to standardize and support evidence based care for common urgent clinical conditions treated in the TASH-ED.

**Table 2:** TDF domains and barriers/facilitators identified, with example quotes

Relevant TDF Domain	Barrier/Facilitator	Example Quote
Knowledge	Lack of awareness of/experience using EBCAs	“I have heard of (EBCAs) but i do not have a deep understanding off it”
Skills	Skill development essential to adoption of EBCAs	(in deciding whether or not to use an EBCA) “I decide whether I am comfortable with the procedure or not”
Social/professional roles and identity	Importance of leadership stressed	“If its accepted by the MOH and hospital we will use”
Beliefs about capabilities	Confidence in ability of TASH-ED to employ EBCAs given tailored to context	‘“everyone can use them” “every discipline should be involved”
Optimism	Continued investment by the Ministry of health will improve resources	“the concern (interest/investment) of the government is now good” “they are building a new ED”
Beliefs about consequences	Use of EBCA will improve efficiency and may improve allocation of resources to ED Expectation of benefits for patient and/or provider important facilitator	“keeping the algorithm in mind makes me efficient” “they (hospital and ministry of health) will want to follow the guideline... They will avail the materials and human power” “It may help us from providing unnecessary medication. And even may help us to save the patient’s life” “It make life easy”, “if I read from the EBCA, I feel confident”
Reinforcement	Belief that making use of the EBCA a requirement and use of sanctions may be needed for staff reluctant to adopt EBCA	“we may not easily take the protocol. To take time, (we) have to be enforced to use” “we make sure the nurses are using the algorithm by making them sign it... monitor and follow-up”
Memory, attention, decision processes	EBCA will act as a memory aid/reminder EBCA should be easy to memorize	“(EBCA helps) something will not be forgotten” “clear and easy to memorize”
Environmental context and resources	Many EBCAs poor fit to TASH-ED resource context Computer/information technology access limited Lack of human resources and high turnover of resources important barriers	“there are medications we don’t have, we don’t have the resources” “we have to base it on what we have here.” “hard to access them. Internet down” “processes/ways of working, and lack of nursing staff (are barriers)” “I think a barrier would come from trainees from other departments... Won’t want to use unless comes from their department”
Social Influences	Group norms and modelling by senior clinicians important to EBCA uptake/use	“whether my peers are using it.. it is accepted by the speciality in general, and other specialities” “I want to see the seniors are doing it, accepting it”
Behavioural regulation	Hard to break habits, new discipline fewer habits to break	“There are some human factors. It is difficult to change what they are used to doing” “it is a new specialty, there is a lot of enthusiasm to develop new ideas, they are very creative”

## Discussion

To our knowledge this is the first study to assess barriers and facilitators to EBCA utilization in a low- and middle- income country (LMIC) ED setting using direct observation and a document search/review to supplement participant reports. While many of the findings of the present study are in keeping with barrier/facilitator assessments of implementation in general and in LMIC in particular, this study provides important information on the relative importance of common barriers/facilitators and identified lack of acceptance of EBCAs outside the ED as a unique barrier of particular importance in the TASH-ED setting. While lack of resources is identified in the majority of barrier/facilitator studies, in high income countries this is often described as lack of time or heavy workload, and to a lesser degree lack of human resources, as noted by Fischer et al. in their scoping review of barriers to implementation which included 69 articles based predominantly on studies conducted in high income countries [9]. In contrast, in keeping with our findings where use of published EBCAs is limited by resources available, barrier/facilitator studies conducted in LMICs commonly reported facing both more significant human resource challenges and often lack of more basic material resources, such as guideline recommended medications and equipment necessary for implementation. For example, our previous study of barrier/facilitator assessments across 5 LMICs found human and material resources including basic medications and equipment key barriers to implementation of evidence [6].

The importance of strong leadership emphasized by participants in our study, has also been highlighted in several barrier/facilitator studies in low/middle-income country studies. Stokes et al.’s systematic review of qualitative evidence from 5 sub-Saharan African countries also identified strong local clinical leadership as a key enabler to implementation of obstetric guidelines [10]. Similar to Vogel et al. in which buy-in was reported as an important facilitator to implementation in several included barrier/facilitator assessments [11], participants in the present study felt that engagement across the range of stakeholders within the TASH-ED would be important to buy-in, uptake and use of EBCAs.

The importance of design, ready accessibility of evidence in the workplace and applicability of EBCAs noted by participants in our study are in keeping with the findings of Fischer et al.’s scoping review [9] which identified the same three factors, and with ease of access arising in several additional barrier/facilitator assessments across a variety of settings [6]. Similarly, lack of awareness or understanding of evidence tools, noted among the majority of participants in the present study, has been identified as an important obstacle to EBCA use in variety of studies [6, 9]. Several findings are uncommonly reported or unique to the present study. Political commitment identified as a potential facilitator in one of the five studies included in our previous study [6], was noted to be important in the TASH-ED context, with recent investment in and support of emergency medicine by the Ethiopian government an important facilitator to EBCA uptake and utilization. Additionally, the recognized need for wide stakeholder engagement and inclusion of representatives from outside the ED were noted as essential facilitators to EBCA development and uptake in the TASH-ED. Barriers identified through direct observation of patient care in the TASH-ED specifically, lack of physical space resulting in crowding and limitation of movement of staff and delays in accessing necessary medications and supplies due to processes of care, are uniquely highlighted in the present study as important barriers to address in implementation planning.

### Strengths & Limitations

Interview participants include the range of both training and experience of staff regularly working in and/or providing essential services to the TASH-ED, allowing for identification of barriers/facilitators from a range of perspectives improving overall understanding of key factors to address in EBCA development and implementation planning, and potential approaches to doing so. Use of multiple data sources including interviews with practicing Toronto ED physicians with experience in working with EBCAs in a variety of clinical contexts and direct observation, provides a comprehensive understanding of identified barriers, facilitators & considerations for EBCA and implementation that would not have been identified through the more common approach to barrier/facilitator assessment employing interviews or surveys alone.

Due to the relatively unique nature of the TASH-ED as the location of the first EM training site and leaders in the development of EM in Ethiopia, the findings from the present study are relatively less generalizable to settings with less developed EM services both within Ethiopia and in other LMICs. In addition, recent improvements in the TASH-ED structure and changes to both structure and function of the ED once the department relocates to the new ED currently under construction, will require reassessment of the structural and process barriers identified in the present study.

## Conclusion

This study found a perceived need for EBCAs tailored to the TASH-ED setting to support uptake of evidence based care into routine practice for common clinical presentations. Barriers and facilitators identified provide information essential to development of both context appropriate EBCAs and plans for their implementation in the TASH-ED. The findings of this work and a prior study, which identified high mortality conditions in the TASH-ED, have helped to prioritize clinical conditions which are likely to benefit from EBCAs and suggest targets for development and implementation of EBCAs tailored to the TASH-ED context.

## Additional file

Additional file 1: Interview Guide. (PDF 52 kb)

## Abbreviations

ACLS: Advanced cardiac life support
ATLS: Advanced trauma life support
EBCA: Evidence based clinical algorithm
ED: Emergency Department
EM: E0mergency Medicine
LMIC: Low- and middle- income countries
SOP: Standard operating procedures
TAAAC-EM: Toronto Addis Ababa Collaboration in Emergency Medicine
TASH: TikurAnbessa Specialized Hospital
