# Supplementary material for: Barriers to and facilitators of the development and utilization of context appropriate evidence based clinical algorithms to optimize clinical care and patient outcomes in the Tikur Anbessa emergency department: a multi-component qualitative study
Source: BMC Health Serv Res. 2019 Mar 20;19:181. doi: 10.1186/s12913-019-4008-2 (PMC6425575; doi:10.1186/s12913-019-4008-2)
Supplement: Supplementary file 1 — Interview Guide. (PDF 52 kb) [file 12913_2019_4008_MOESM1_ESM.pdf]

Additional file 1: Interview Guide

Before we begin I want to remind you of a few important points. The interview is expected to last 30 to 60 minutes and will be audio-recorded. Your participation is voluntary and you may refuse to answer any question or withdraw from participation at any time without penalty. Also, no personal information or responses will be released to anyone outside the study team at any time, and your responses will be coded so that they cannot be identified. Your participation or non-participation in this study will therefore not affect your current or future employment status or performance appraisal.

Discussion Guide:

1. *Do you have any questions?*
    If yes, answer questions.
    If no, may we proceed with the interview now then?

2. *First I would like to ask you a few questions about yourself.*
    Gender M/F
    Age ___
    Current Employer:
        Tikur Anbessa Specialized Hospital (TASH ED) _____
        University of Toronto Affiliated Hospital ______
    Current Job at Tikur Anbessa Hospital:
        Clinical Staff: Physician/Nurse/Other _______________
            For clinical staff: level of training & specialization____________________
        Administrative Staff: Please describe role_______________
        Support Staff: Please describe role_____________________
    Years at Tikur Anbessa Hospital____________
    Years in current position _______________
    UofT EM physicians: Total number of months worked clinically in the TASH -ED___________

3. *Have you heard of Evidence based clinical algorithms, such as clinical practice guidelines, clinical pathways, order sets, and clinical decision rules. Yes/No*
    *If yes, what have you heard/do you know about them*_____________________

**** regardless of answer above, please define EBCAs before going to next question***
    "EBCA are tools to facilitate the incorporation of evidence into routine clinical practice by putting evidence into specific rules or action plans that can be used for patient care."

4. Do you have any experience with EBCA? Yes/No
    *If yes, please describe*________________________________

5. *Are you aware of any EBCA that are used in/or available for use in caring for patients in the TASH-ED*
    If yes, what guidelines/tools are available: _________________ *(include who tool is produced by)*
    *Which is any do you use/have you seen used:*____________________________________
    *How often do you use them/have you seen them used:*_____________________________
    If used, are they helpful? Why/why not?
    *If aware/available but not used, why?*____
        *Probes: if concerns with quality/utility of tools, ask to explain.*____________
            *If resource limitations noted as reason, ask to specifiy*____________
            *If not consistent with roles/responsibilities, ask to explain*______________
            *If conflicting guidelines, ask to explain further*_______________________

6. *Do you think having context appropriate EBCAs for common clinical presentations seen in the TASH-ED would be helpful?*

*Why or why not?*______________________________

7. If TASH wanted to develop context appropriate EBCAs for the emergency department, what clinical presentations do you think they would be most helpful for, and why?__________________________

8. What would need to happen/be in place for a new EBCAs developed for the TASH-ED to be implemented?____________________

9. Are you aware of any barriers/challenges to implementing a new EBCA in the TASH-ED?_________________________

Probes for barriers:

- at system level: (roles/responsibilities/regulations, workplace norms/culture, resources)
- provider level (knowledge, attitudes, roles/responsibilities)
- patient level (resources, culture/belief systems)

10. Are you aware of any factors that might facilitate uptake/implementation of EBCAs?__________

Probes for Facilitators:

- at system level (roles/responsibilities/regulations, workplace norms/culture, resources) - provider level (knowledge, attitudes, roles/responsibilities)

- patient level (resources, culture/belief systems)

11. In making a decision about whether or not to adopt a new procedure at work in the TASH-ED, what factors are most important to your decision?___________________

What do you think is most important to your colleagues?____________________

12. Is use of EBCAs compatible with/in conflict with professional roles/responsibilities,

work place norms ____________

- Is this the case for all clinicians who work in the TASH ED ____________________

13. *Do you feel that it is appropriate for patient care to be based on EBCAs? why or why not?*______________________________

*- how do you think others in your group/other clinical groups feel about the use of EBCAs for patient care?*______________

14. Are there any resources, processes or ways of working that would be need to change or that would promote use of EBCAs in the TASH ED________________________________

15. If new knowledge or skills were required to implement an EBCA, how should this be addressed*?*_____________________

16. *Do you have anything you want to add or clarify?*

17. *Do you have any questions?*

18. Thank you for your time.
